# Pacing lead thrombus in patient with recent COVID-19 infection and subsequent vaccination: a case report

**DOI:** 10.1093/ehjcr/ytae447

**Published:** 2024-08-23

**Authors:** Alberto Palazzuoli, Christian Mingiano, Niccolò Manetti, Chiara Leolini, Antonella Fossi

**Affiliations:** Cardiovascular Diseases Unit, Cardio Thoracic and Vascular Department, Le Scotte Hospital, University of Siena, 53100 Siena, Italy; Department of Medicine, Surgery and Neurosciences, University of Siena, 53100 Siena, Italy; Cardiovascular Diseases Unit, Cardio Thoracic and Vascular Department, Le Scotte Hospital, University of Siena, 53100 Siena, Italy; Cardiovascular Diseases Unit, Cardio Thoracic and Vascular Department, Le Scotte Hospital, University of Siena, 53100 Siena, Italy; Respiratory Disease Unit, Department of Medical Sciences, University Hospital of Siena, Siena, Italy

**Keywords:** Case report, Cardiac COVID-19 complication, Intracardiac thrombosis, Pulmonary embolism, Intracardiac device, Vaccine side effects

## Abstract

**Background:**

The association between acute coronavirus disease-19 (COVID-19) infection and a hypercoagulable state has been exhaustively described throughout the pandemic. The presence of external devices, such as intracardiac leads, could predispose to higher thrombotic risk in this setting. We present a clinical case of intracardiac thrombosis on right ventricle device that occurred after COVID-19 infection and subsequent vaccination.

**Case summary:**

A 56-year-old man, suffering from usual interstitial pneumonia-pattern fibrosis, was admitted to our hospital because of worsening of his clinical status. About 10 days earlier, he had got vaccinated for COVID-19. Three months earlier, the patient had been reported to have severe acute respiratory syndrome coronavirus 2 (SARS-CoV2) infection. A chest computed tomography scan showed thrombus partially occluding the left pulmonary artery. A transthoracic echocardiography and later a transoesophageal echocardiogram showed a mass adhered to the lead in the right ventricle, compatible with thrombosis, confirmed on a cardiac computed tomography scan. Blood tests showed no major changes except for a slight increase in D-dimer and fibrinogen. Therefore, the subject was treated with anticoagulants.

**Discussion:**

COVID-19 infection results in a hypercoagulable state with risk of developing thrombus diffusely, including intracardiac thrombosis. The presence of external devices, such as the intracardiac leads, may increase thrombotic risk since the presence of an external device in the bloodstream could trigger coagulation cascade. This case report highlights the need for special care in this patient setting, using specific imaging techniques for early and rapid diagnosis to optimize therapy.

**Learning points**

- To highlight thromboembolic complications (including both pulmonary embolism and intracardiac thrombosis) in individuals with cardiac device after coronavirus disease-19 (COVID-19) infection with previous vaccination.
- To focus on late diagnostic management after COVID-19 infection and consequent antithrombotic therapy during post-discharge acute phase in subjects with intracardiac device.
- To provide an alert on early vaccine administration in patients with intracardiac device after acute COVID-19 infection.

## Introduction

Severe acute respiratory syndrome coronavirus 2 (SARS-CoV2) pandemic showed that infectious diseases can exacerbate a state of hypercoagulability. The association between coronavirus disease-19 (COVID-19) and subsequent early vaccination for that infection could even increase thrombotic risk. We present the first report describing a clinical case of intracardiac thrombosis on right ventricle device that occurred after acute COVID-19 infection.

## Summary figure

**Figure:**
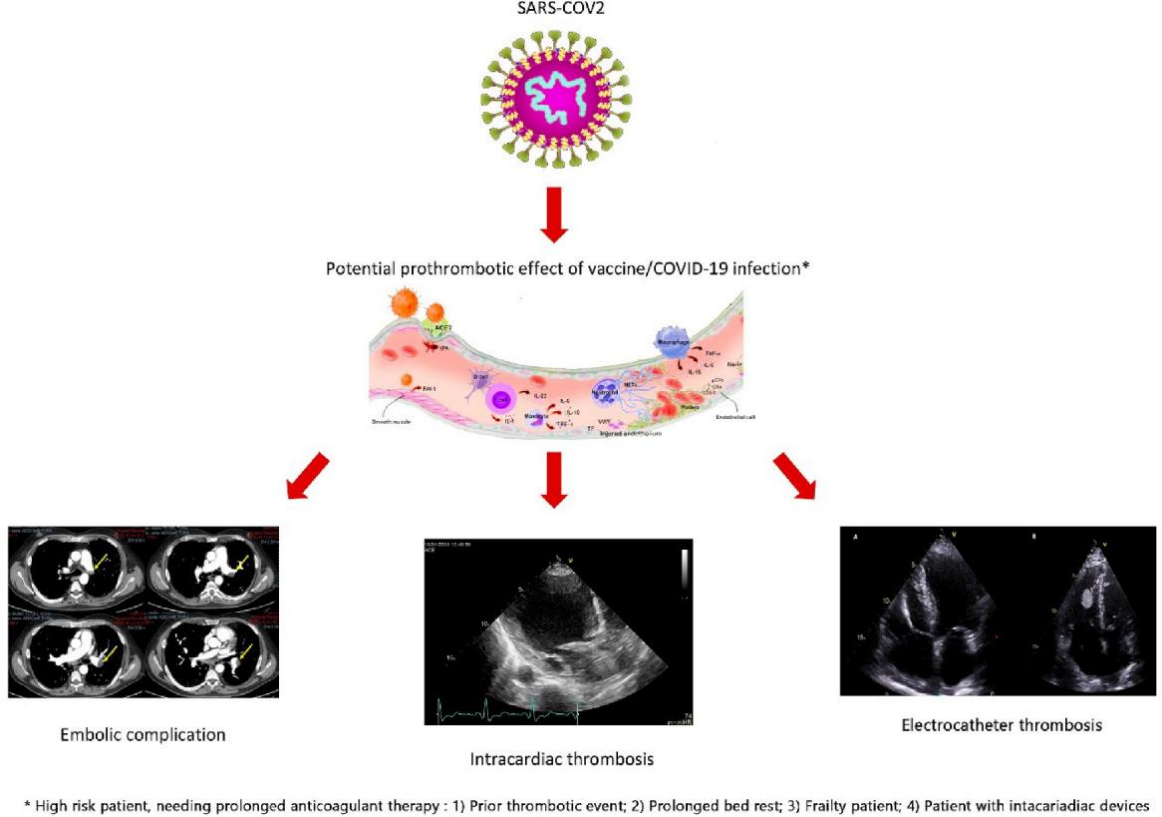
Thrombus intracardiac device occurrence after vaccine administration: a potential complication leading to pulmonary embolism.

## Case presentation

A 56-year-old man was admitted to our hospital because of worsening dyspnoea and reduced exercise tolerance. The subject was affected by usual interstitial pneumonia-pattern (UIP) fibrosis. The physical examination showed rare crackles in the inferior lung fields. Ten days before hospital admission, the patient had received vaccine for COVID-19. Three months earlier, the patient had been diagnosed with COVID-19 infection without hospital admission. Few years earlier, he underwent dual-chamber rate-modulated pacemaker (DDD-R Astra MEDTRONIC) implant because of repetitive syncope episodes with sinus node dysfunction. The patient had received COVID-19 vaccinations in the previous year.

**Figure 1:**
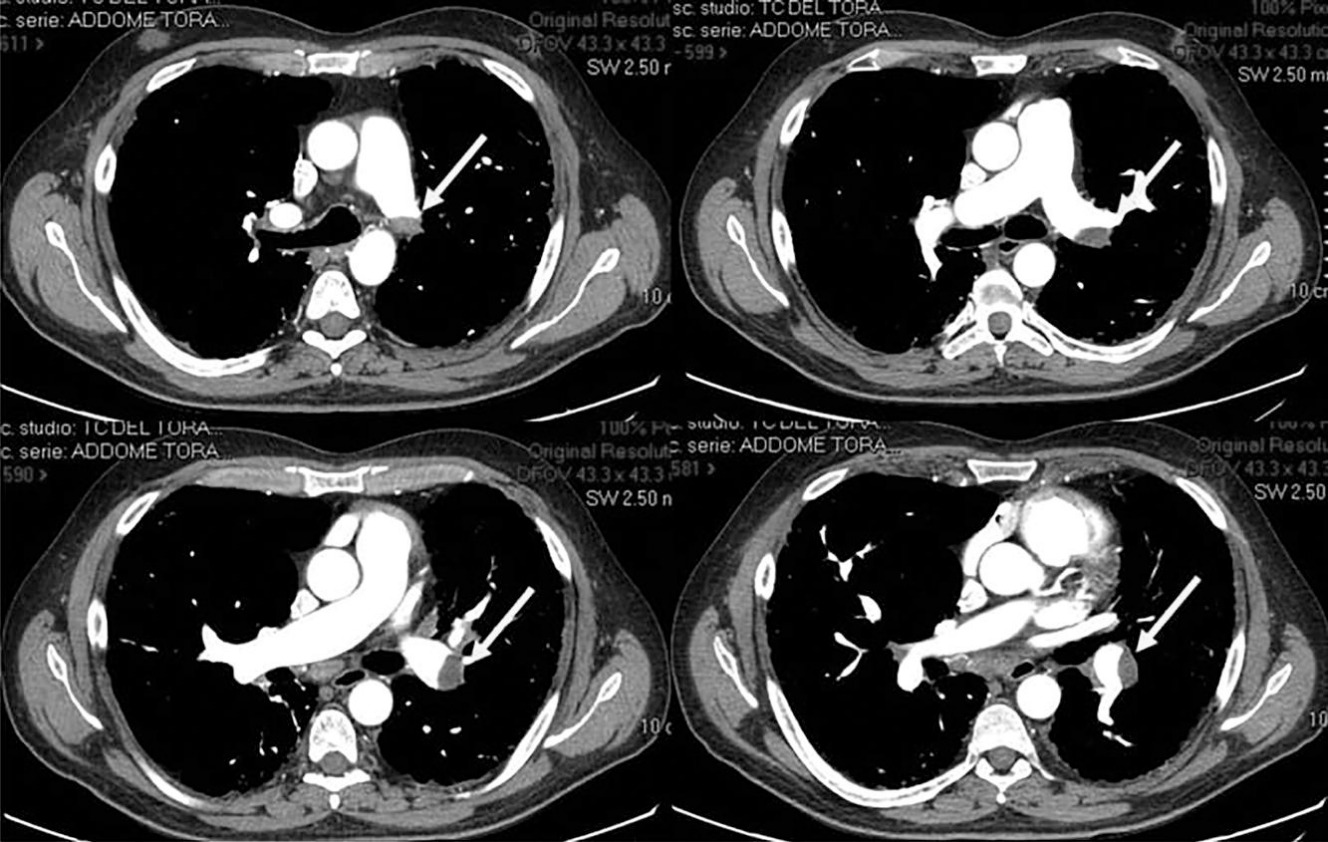
Computed tomography scan of the chest. After administration of contrast medium, partially occluding thrombosis was shown in left pulmonary artery and its main branches.

**Figure 2:**
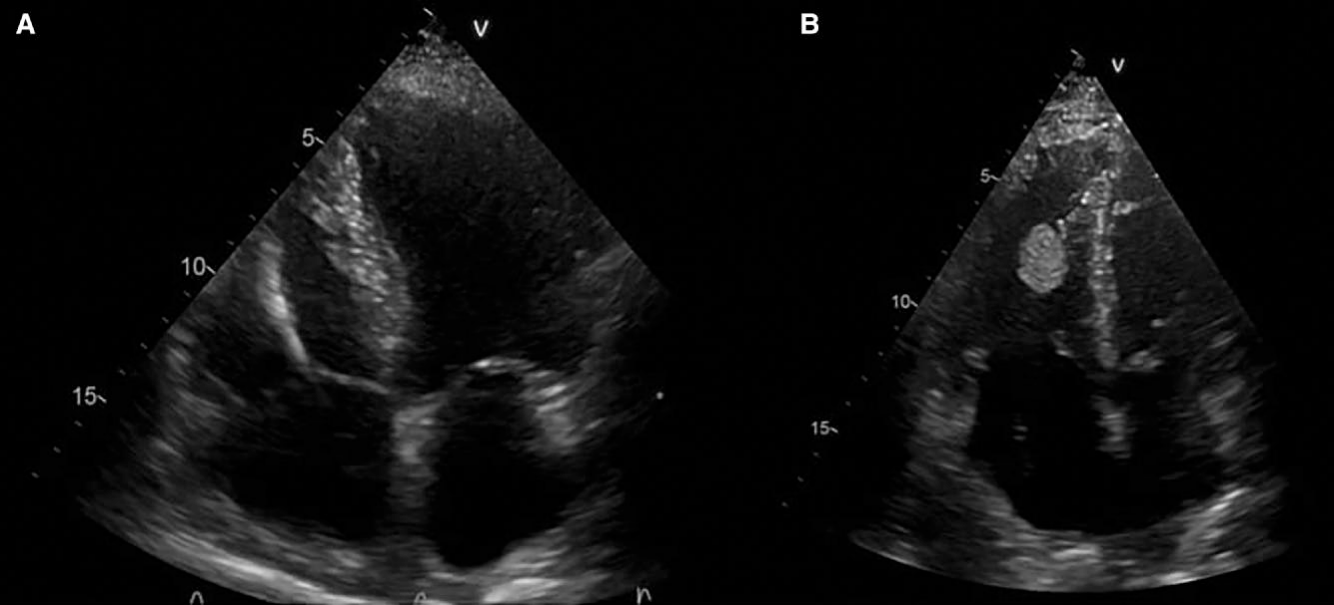
Transthoracic cardiac echocolordoppler a year before the diagnosis of intracardiac thrombus (*A*). Transthoracic cardiac echocolordoppler showing thrombus on pacing lead of right ventricle (*B*).

**Figure 3:**
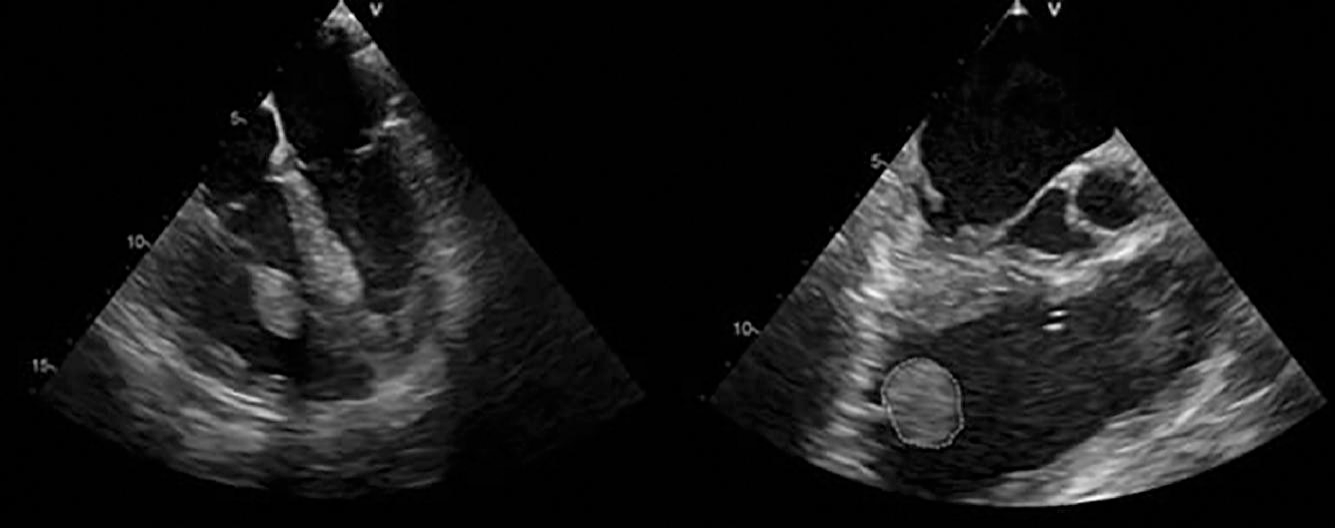
Transoesophageal cardiac echocolordoppler showing thrombus on pacing lead in the right ventricle (area 2.9 cm^2^).

**Figure 4:**
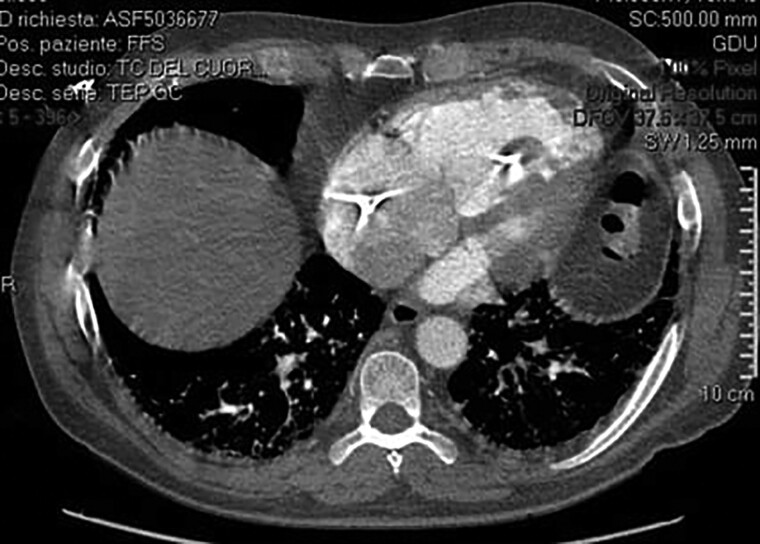
Computed tomography of the heart. A voluminous mass (area 2.6 cm^2^) on right ventricle pacing lead.

During hospitalization, the patient underwent computed tomography (CT) scan of the chest showing partially occluding thromboembolism in the left pulmonary artery and its segmental branches (*Figure 1*). A transthoracic cardiac echocolordoppler (TTE) showed the presence of a hyperechogenic, roundish, clear-edged mass adhered to the pacing lead into the right ventricle (*Figure 2*). The transoesophageal echocolordoppler (TEE) showed a voluminous ellipsoidal shape mass (area 2.9 cm^2^) compatible with thrombosis adhered to the terminal portion of the pacing lead (*Figure 3*). These findings were confirmed by a cardiac CT scan (*Figure 4*). Therefore, screenings for autoimmune diseases, acquired coagulopathies, and thrombophilia (prothrombin mutation, antithrombin III, antiphospholipid antibodies, lupus anticoagulant, homocysteine, activated protein C resistance, qualitative and quantitative C- and S-protein) were performed, all of which were negative. Patient also underwent Doppler venous examination, which excluded a deep venous thrombotic process. Moreover, an endocarditic vegetation was excluded by a negative clinical and laboratory setting (negative inflammatory markers and normal white blood count, absence of fever and other clinical signs of endocarditis); two consecutive blood culture, negative for bacterial and other fungi growing. Other laboratory investigations showed an increase in D-dimer and fibrinogen levels, consistent with an ongoing hypercoagulability state.

In relation to the clinical presentation, the patient had an intermediate pulmonary embolism (PE) risk, so according to the risk-adjusted management strategy, he underwent endovenous anticoagulant therapy with intravenous unfractionated heparin for 7 days. The activated partial thromboplastin time (APTT) was maintained upper 60 ms; during the endovenous administration treatment, imbrication with oral anticoagulant warfarin was performed, maintaining INR between 2 and 3, as suggested by the pulmonary embolism and by intracardiac thrombosis recommendation guidelines.^1^ The choice of this anticoagulant therapy was mandatory, since the direct oral factor X and direct thrombin antagonists do not demonstrate efficacy on acute intracardiac thrombus resolution. Because of concomitant cardiac thrombosis associated with PE, we chose to prolong the administration of warfarin beyond the recommended 3 months period for high-intermediated risk profile.^1^

**Figure 5:**
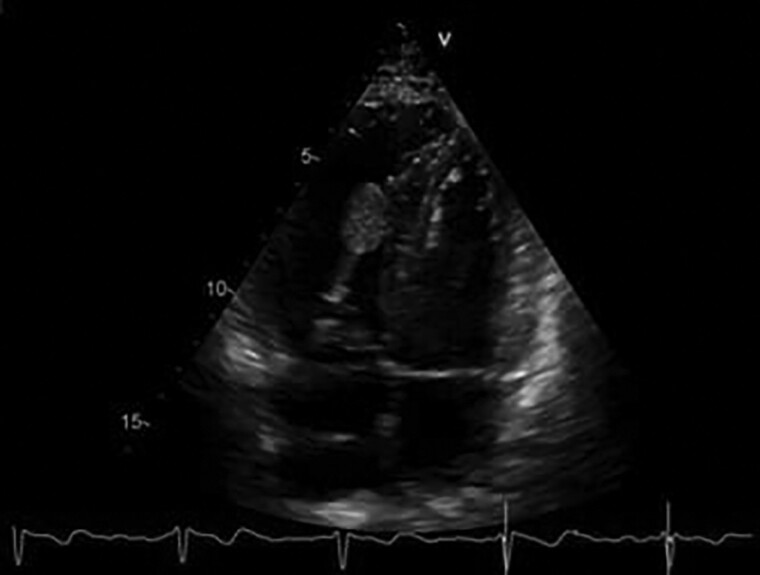
Transthoracic cardiac echocolordoppler showing the reduction of thrombus area on pacing lead in right ventricle (area: from 2.9 to 2.0 cm^2^) after 1 week of anticoagulant therapy.

The TTE performed after 1 week of heparin administration showed a slight reduction in size of the endocavitary mass (area from 2.9 to 2.0 cm^2^; *Figure 5*). The patient is currently in follow-up and he continues the anticoagulant treatment. He is still under evaluation by regular pneumology and cardiology checks up every 6 months.

## Discussion

Several clinical cases reported a higher risk of intracardiac thromboembolic complications associated with COVID-19. However, this is the first clinical case of thrombus that occurred on intracardiac pacing lead, which subsequently caused pulmonary embolism. Although we cannot definitely exclude the onset of intracardiac thrombus related to the primitive COVID-19 infection, because the patient was not hospitalized for primitive infection, during the diagnostic screening and check-up performed on him (TTE, CT scan), no evidence of intracardiac thrombosis was found. Current findings and clinical history strongly suggest the onset of clot was mainly related to the subsequent vaccination.

The association between COVID-19 infection and alteration of the coagulation cascade was exhaustively described during the pandemic.^2^ Notably, several autoptic examination after COVID-19 death documented pulmonary embolism and deep vein thrombosis, without *in vivo* diagnosis.^3^ Similarly, the infection also induced microvascular thrombosis in the perialveolar district, endothelial dysfunction, and diffuse inflammatory cell invasion.^4^

The mechanisms underlying the dysfunction of the coagulation cascade are multiple. A key role is attributed to endothelial dysfunction, which is associated with oxidative stress and increased permeability, cytokines storm, increased of collagen activity, and overexpression of prothrombotic factors.^5,6^ Moreover, the SARS-CoV-2 infection itself induced overproduction of pro-inflammatory cytokines by the interaction with the innate immunity, which leads to a prothrombotic status.^7^

Another important role is probably driven by the interaction between COVID-19 and angiotensinogen-converting enzyme-2 (ACE-2) receptor, widely expressed on epithelial cells and pulmonary vessels. This interaction leads to an increased bioavailability of angiotensin II, which in turn causes platelets to adhere to the endothelium resulting in platelet aggregation and thrombus formation.^7^

Alteration of laboratory parameters related to coagulation status represents one of the main negative prognostic factors regarding COVID-19: prolonged prothrombin time (PT) and APTT and increased serum D-dimer and fibrinogen values, factor VIII and Von Willebrand factor, which may be suggestive for increased thrombotic risk.^8,9^ However, our patients did not present specific laboratory marker alteration neither autoimmune disorder excepting for mild D-dimer level elevations that may be suggestive of an acute thrombotic state. Current literature did not provide sufficient evidence regarding the risk of PE and intracardiac thrombosis following COVID-19 and mRNA vaccination: in a retrospective cohort study, Harris *et al.*^10^ did not find increased risk related to vaccination, including pulmonary embolism and thrombocytopenia and myocarditis, across 1 month of follow-up period. Conversely vaccine-induced thrombotic thrombocytopenia is a common mRNA vaccine complication associated with disseminated intravascular thrombosis, including stroke, pulmonary embolism and splanchnic and cerebral venous thrombosis.^11^ Current contrasting findings do not support a universal antithrombotic strategy. Since any case description of pacing lead thrombosis has not been reported, we treat the patient with warfarin, considering a similar risk to native intracavitary ventricular thrombus. The only multicentre study including data about intracardiac clot revealed a strict relationship with persistent atrial fibrillation and did not provide specific recommendation regarding the anticoagulation treatment timing and regimen to be done. Indeed, the observed patients had a very different time period of anticoagulant administration.^12^

Our clinical case represents the first case in the literature concerning a thromboembolic event on a pacing lead resulting in pulmonary embolism in a subject with a hypercoagulable state due to a prior COVID-19 infection. With this clinical case, we would alert that after acute infection resolution, vaccination may enhance the risk of thrombosis and hypercoagulability status even without a clear laboratory alteration. Physicians may be aware that subjects with inflammatory diseases and pacing lead are much more prone to develop thromboembolic complications. In this regard, there are no specific indications on the correct follow-up. Nevertheless, it could be worth to achieve a stricter diagnostic follow-up by TTE and specific coagulation blood tests (i.e. D-dimer, fibrinogen, PT, APTT) across a prolonged period.

In conclusion, COVID-19 infection may cause a state of hypercoagulability in patients with lung disease, even after acute phase resolution when vaccination is administered during early post-COVID-19 period. The presence of devices, such as pacing lead, may increase thrombotic events. With this case, we would highlight the matter of advanced imaging application to identify cardiac and pulmonary thromboembolic processes throughout the infection period (acute and post-discharge phase). This screening should be performed to early detect an ongoing thrombotic process and eventually begin an appropriate antithrombotic management. Therefore, independently of laboratory findings and thrombotic status, patients with systemic pulmonary diseases and those with intracardiac device deserve careful diagnostic monitoring to identify patients with potential risk.

## Lead author biography


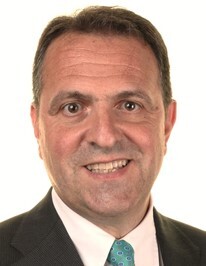


Alberto Palazzuoli is an Italian cardiologist with vast research activity, primarily focused on heart failure treatment and the impact of comorbidities in heart failure, with special attention to renal dysfunction. He is also involved in research activity on cardiovascular imaging. He is credited as chair of the SIC group of cardiac insufficiency, and he is one of the researchers who coined the term cardiorenal syndrome. He is currently FESC FEACVI and FHFA member. He is into the HFA board of imaging in heart failure study group.

**Consent:** The patient gave written consent at the hospital admission, for the treatment of the personal data. Personal clinical information is managed according to the COPE guidelines, and all the images presented in this article have been anonymized, in order to respect privacy and sensible data.

**Funding:** None declared.

## Data Availability

Data are available on request.
